# Research on Ultrasonic Focusing Stacked Transducers for Composite

**DOI:** 10.3390/s25196179

**Published:** 2025-10-06

**Authors:** Yi Bo, Jie Li, Shunmin Yang, Chenju Zhou, Yutao Tian

**Affiliations:** School of Instrument and Electronics, North University of China, Taiyuan 030051, China; sz202306068@st.nuc.edu.cn (Y.B.); ysmsoft@nuc.edu.cn (S.Y.); sz202306063@st.nuc.edu.cn (C.Z.); s202506074@st.nuc.edu.cn (Y.T.)

**Keywords:** focusing stacked transducers, narrow-pulse ultrasonic transducers, carbon fiber composites

## Abstract

Most existing carbon fiber composite materials are formed by high-temperature molding of multiple layers of fiber cloth. During the manufacturing and usage processes, materials are prone to defects such as voids, delamination, and inclusions, which seriously threaten their service life and safety performance. Ultrasonic testing is currently a widely adopted method for detecting defects in carbon fiber composite materials. However, existing narrow-pulse ultrasonic transducers often have to sacrifice emission energy to achieve narrow-pulse emission, which results in their limited ability to penetrate thicker carbon fiber composite materials. To address this issue, this paper proposes the design of a focused laminated transducer. By stacking and bonding lead titanate piezoelectric wafers and using a concave lens made of organic glass to focus ultrasonic waves, the emission sound intensity of the ultrasonic transducer is enhanced. The simulation results show that the designed focused double-stack transducer has a directivity gain that is 4.49 dB higher than that of the traditional single-piezoelectric-wafer transducer. The transducer fabricated based on this design has successfully achieved effective detection of internal defects in carbon fiber composite materials.

## 1. Introduction

### 1.1. Purpose and Significance

Carbon fiber composite materials are composed of carbon fibers and matrices, such as epoxy resin, and are a new type of fiber material with a carbon content exceeding 90%. Recently, composite materials, especially carbon fiber-reinforced polymer composites (CFRPs), are continuously ousting different orthodox metal and metallic alloys owing to their possession of low specific gravity, better strength, higher stiffness, facile fabrication process, higher corrosion resistance, improved fatigue resistance, extended life cycle property, and, most importantly, convenient-lightweight structure [1,2,3,4,5,6,7,8].

Carbon fiber composite materials are mainly formed by compression molding. Compression molding is a forming method in which carbon fiber pre-impregnated materials are placed in molds, and the resin is cured by applying pressure and temperature to obtain products of the desired shape and performance. The compression molding process begins by cutting the carbon fiber pre-impregnated material according to the design requirements and laying it in a mold made of metal or alloy with a specific shape and size. Then, the mold is placed on a hydraulic press or hot press, and by applying certain pressure and temperature, the resin is fully impregnated and cured in the carbon fiber [9]. Due to the precision of mold processing and the stability of the technology, this process can produce carbon fiber products with stable performance and precise dimensions, while also enhancing production efficiency and achieving automated production. However, during the processing and application of this technology, carbon fiber composite materials have anisotropy and are prone to defects such as voids, inclusions, cracks, and delamination. As the stress of carbon fiber composite laminates continuously spreads during use, the residual strength of structural components significantly decreases, which may lead to overall structural failure or other catastrophic consequences [8,10]. Therefore, the application of carbon fiber composite materials needs to be evaluated by non-destructive testing methods.

A wide range of non-destructive testing applications, including ultrasonic testing, are successfully used to analyze defects in composites [11,12,13,14]. There is a prerequisite for the commonly used non-destructive testing methods in composite material inspection: the problem morphology must first be determined through damage detection and then compared with the non-destructive testing results to determine the existence, location, size, and quantity of the defects. The commonly used non-destructive testing methods at present include X-ray (radiation method), ultrasonic (acoustic method), and CT (electromagnetic method) [15].

X-ray inspection technology is a traditional non-destructive testing technique that is applied in various fields. This detection technology detects whether there are defects in the object under test through the penetrating power of X-rays [16]. X-ray presentation detection technology mainly includes real-time X-ray imaging technology (photographic technology, DR Technology, and CR technology) and CT imaging detection technology (tomographic CT and micro-CT). Its advantages include the ability to conduct qualitative and quantitative analysis of defects, intuitive defect display, high detection accuracy, and no restrictions on the shape of the test piece [17]. However, X-rays are quite harmful to the human body. If used for a long time, they can cause certain damage to the body and may not detect delamination defects. At the same time, its low detection efficiency, high detection cost, and low detection thickness also limit its application in industrial non-destructive testing.

Eddy current testing (ECT), as one of the widely used electromagnetic non-destructive testing (NDT) methods, is applied to inspect key structures in the energy, aerospace, and many other industries due to its non-contact, high sensitivity, and low cost features [18]. Eddy current testing is based on electromagnetic induction. When the detection coil is brought close to a conductive carbon fiber plate cable specimen, a closed-loop eddy current flows within it, and the magnetic field generated by the eddy current acts on the detection coil again, causing impedance changes [19]. Eddy current testing is a non-contact detection method, featuring no need for coupling agents, high sensitivity, and rapid scanning. However, its drawbacks are also quite obvious. Eddy current testing is only suitable for surface defect detection, requires complex signal interpretation, has high requirements for material surfaces, and is associated with high costs.

Ultrasound detection technology is a very well-known, effective technique used in non-destructive testing (NDT) [20]. Ultrasonic non-destructive testing mainly utilizes the propagation characteristics of ultrasonic waves in composite materials. By recording the changes such as scattering, attenuation, reflection, and resonance of ultrasonic waves in the material under test, it analyzes the existence of defects in the material. When conducting ultrasonic testing, there are usually sensor probes responsible for transmission and reception. According to the position of the probe during ultrasonic testing, ultrasonic testing can be classified into transmission method, reflection method [21,22,23], Rayleigh method, etc. Ultrasonic testing offers advantages such as high resolution, deep defect detection, high stability, and no harm to the human body. At the same time, it can detect the uniformity of materials and tiny cracks. Carbon fiber is composed of multiple materials and has a very high sound attenuation rate. Piezoelectric transducers are an important type of transducer, and people have been conducting relevant research to improve their performance. S. M. Yang, W. A. Song, Y. F. Chen, L. Yang, and others studied a quadrupled ultrasonic transducer for the detection of viscoelastic media. Meng, XD, Lin, and SY [24] designed a cascaded piezoelectric ultrasonic transducer with three sets of piezoelectric ceramic stacks. Wu, SR, Xie, YH, and Bai, FS [25] conducted an optimization design and experimental study on a new type of relaxed ferroelectric single crystal transducer based on the surface shear vibration mode. Zang, XL, Xu, ZD, and Lu, HF [26] fabricated a stacked piezoelectric transducer with a delay layer, which enhanced the performance of ultrasonic transducers. Wen, SH, Xu, L, Gong, T, Zhang, HD, Liang, ZF, and Yao, L [27] studied a longitudinally curved composite axial laminated piezoelectric ultrasonic transducer designed based on the principle of acoustic black holes, achieving the focusing of piezoelectric transducers.

When inspecting composite materials, the noise signal in the ultrasonic signal and the defect echo signal fuse together, making it difficult to distinguish the defect echo and causing difficulties in identifying internal defects of the material. Therefore, narrow-pulse ultrasonic transducers need to be used for detection to distinguish defect echo signals. However, narrow pulses require sacrificing the flaw detection sensitivity of the transducer to achieve this. At this time, the use of lamination and focusing methods can effectively increase the sound intensity of the ultrasonic transducer. This article proposes the design of a 2 MHz focused ultrasonic laminated transducer, which ensures narrow pulses while maintaining the amplitude of the signal.

### 1.2. Resonance Frequency

In cylindrical coordinates, the motion equation for a single piezoelectric wafer is

(1) $$\rho \frac{\partial^{2}\zeta_{r}}{\partial t^{2}}=\frac{\partial T_{r}}{\partial r}+\frac{T_{r}-T_{\theta }}{r}$$

where r is the radius, ρ is the density of the piezoelectric wafer, $\xi_{r}$ is the radial displacement component, t is the thickness, $T_{r}$ is the radial stress component, and $T_{\theta }$ is the circumferential (hoop) stress component.

Since the electric field is applied along the z-axis, and the boundary effects of the electric field are neglected, the electric field component $E_{z}$ is treated as uniform. The piezoelectric constitutive equations simplify to

(2) $$\begin{array}{l} S_{r}=s_{11}^{E}T_{r}+s_{12}^{E}T_{\theta }+d_{31}E_{z}\\ S_{\theta }=s_{12}^{E}T_{r}+s_{11}^{E}T_{\theta }+d_{31}E_{z}\\ D_{z}=d_{31}T_{r}+d_{31}T_{\theta }+\varepsilon_{33}^{T}E_{z} \end{array}$$

Here, $S_{r}$ and $S_{\theta }$ represent the normal strains along r and θ. $s_{11}^{E}$ and $s_{12}^{E}$ represent the piezoelectric constants. $D_{z}$ represents the electric field intensity along the z direction, and $\varepsilon_{33}^{T}$ represents a component of the dielectric constant. $d_{31}$ represents a component of the piezoelectric strain constant. We can derive the following from the above equation:

(3) $$\begin{array}{l} T_{r}=(\frac{Y_{0}^{E}}{1-\sigma^{2}})(S_{r}+\sigma S_{\theta })-\frac{d_{31}Y_{0}^{E}}{1-\sigma }E_{z}\\ T_{\theta }=(\frac{Y_{0}^{E}}{1-\sigma^{2}})(S_{\theta }+\sigma S_{r})-\frac{d_{31}Y_{0}^{E}}{1-\sigma }E_{z}\\ D_{z}=d_{31}(T_{r}+T_{\theta })+\varepsilon_{33}^{T}E_{z} \end{array}$$

Here, $Y_{0}^{E}$ a represents the Young’s modulus, and σ=−s12Es11E denotes the Poisson’s ratio.

Similarly, the mechanical vibration equation can be derived as follows:

(4) $$F=j\rho vS[-\frac{J_{0}(ka)}{J_{1}(ka)}+\frac{1-\sigma }{ka}]\xi_{a}+nV$$

Here, F represents the stress applied to the piezoelectric wafer, and ρ denotes the density of the piezoelectric material. $v=\sqrt{\frac{Y_{0}^{E}}{\rho (1-\sigma^{2})}}$ represents the wave propagation speed (acoustic velocity) in the piezoelectric material. S represents the cross-sectional area of the piezoelectric element. $J_{0}(ka)$ a represents the zeroth-order Bessel function of the first kind, and $J_{1}(ka)$ z represents the first-order Bessel function of the first kind. $\zeta_{a}$ represents the resonant velocity. $n=\frac{2\pi ad_{31}Y_{0}^{E}}{1-\sigma }$ represents the electromechanical coupling coefficient. V represents the applied voltage.

Similarly, the following equation represents the circuit state equation:

(5) $$I=j\omega C_{0}V-n\zeta_{a}$$

Here, C0=πa2ε¯33t represents the blocking capacitance.

**Figure 1:**
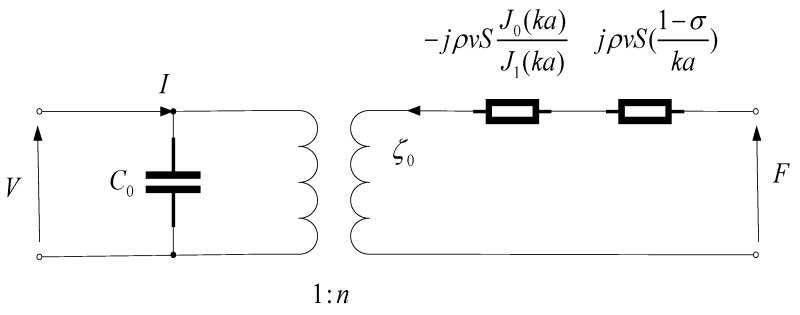
Electromechanical equivalence diagram of a single piezoelectric chip.

Based on Equations (4) and (5), the piezoelectric wafer can be equivalently represented by the electrical circuit shown in Figure 1.

The arrows here indicate the directions of force and voltage, respectively. For a freely vibrating piezoelectric wafer (with no external force, F = 0), the resonance frequency equation of a single piezoelectric element is derived as follows:

(6) $$kaJ_{0}(ka)=(1-\sigma )J_{1}(ka)$$

Therefore, the admittance equation of the piezoelectric wafer is

(7) $$Y=j\omega C_{0}+\frac{n^{2}}{j\rho vS[\frac{1-\sigma }{ka}-\frac{J_{0}(ka)}{J_{1}(ka)}]}$$

**Figure 2:**
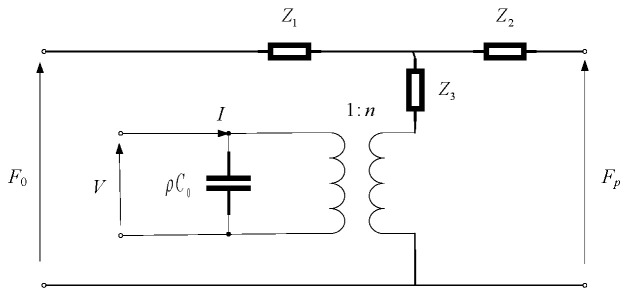
Mechanical equivalent circuit for piezoelectric wafer stacking.

The stacked transducer consists of two piezoelectric wafers, which are connected in parallel to the electrical circuit structure and in series in the mechanical structure. Based on the cascade theory in electrical circuits, the electromechanical equivalent circuit diagram of the stacked transducer can be derived [28,29]. As shown in Figure 2, the admittance of the stacked transducer is the superposition of each piezoelectric wafer’s admittance. Therefore, the admittance of the dual-wafer transducer can be derived as follows:

(8) $$Y=2j\omega C_{0}+\frac{n^{2}}{j\rho vS[\frac{1-\sigma }{ka}-\frac{J_{0}(ka)}{J_{1}(ka)}]}$$

**Figure 3:**
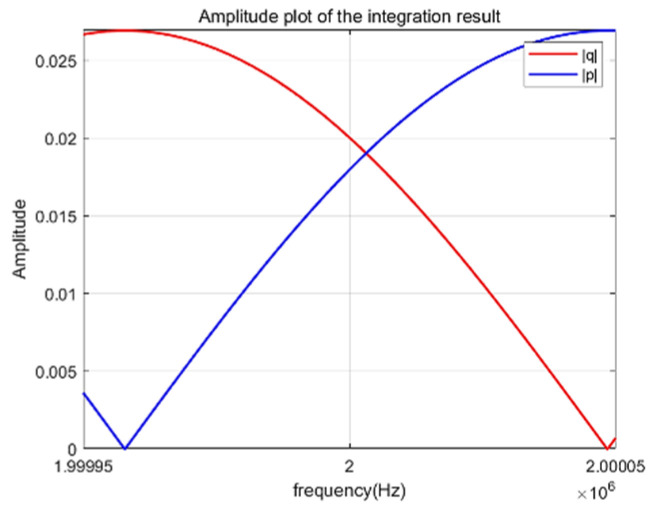
Calculation curves beyond equations.

When Y→∞, the transducer operates at resonance. At this point, the vibration frequency of the transducer is its resonant frequency. Let $\left|p|=kaJ_{0}(ka\right)$ and $\left|q|=(1-\sigma )J_{1}(ka\right)$. We solved the equation and plotted the graph in the range from 1,999,950 Hz to 2,000,050 Hz, as shown in Figure 3.

As shown in Figure 3, the solution of the transcendental equation near 2 MHz deviates only slightly from the ideal 2 MHz point. This implies that a 2 MHz ultrasonic transducer exhibits minimal theoretical error and should perform effectively.

## 2. Design of Ultrasonic Focusing Transducer Scheme Based on Laminated Structure

### 2.1. The Structural Design of the Transducer

**Figure 4:**
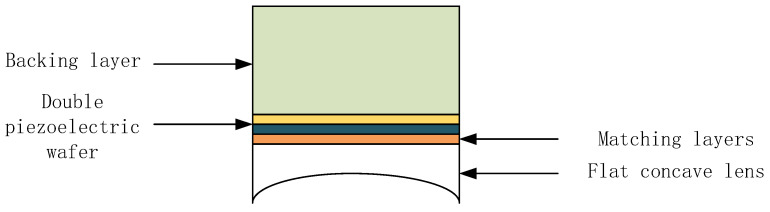
Transducer structure diagram.

The structure of the dual piezoelectric wafer focusing transducer is shown in Figure 4 It consists of a backing layer, a matching layer, piezoelectric wafers, and plano-concave lenses.

**Figure 5:**
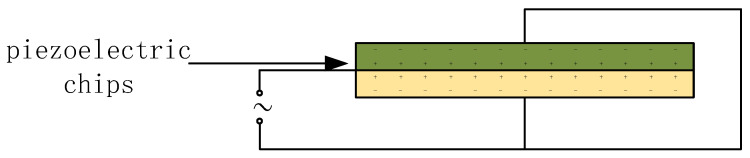
Connection method of piezoelectric wafers.

Piezoelectric wafer: Two wafers have opposite polarization directions and are stacked in the direction of the electric field. The thickness vibration is generated overall when voltage is applied. This thickness mode is more efficient than the thickness expansion mode of a single piece, has a greater displacement, and can effectively suppress unnecessary harmonic vibrations. The piezoelectric wafers are connected in parallel in the circuit, making the two wafers equivalent to a capacitor, and their capacitance values are the same as those of a single wafer. This makes the electrical impedance of the transducer comparable to that of a single chip, making it easy to match the impedance with standard electronic drive circuits without the need for extremely high voltages. The connection method of the piezoelectric wafer is shown in Figure 5.

The backing material has the characteristic of high sound attenuation, which can quickly absorb and dissipate the acoustic energy radiated backward by the wafer, preventing it from being reflected back to the wafer. This significantly reduces the residual vibration of the pulse, resulting in a narrower pulse. At the same time, it can also improve the resolution of the ultrasonic transducer.

The function of the matching layer is to coordinate the difference between the high-sound impedance of the transducer and the dielectric impedance. By means of impedance gradient, it minimizes the reflection loss of sound waves at the interface, thereby significantly improving the transmission rate of sound energy, enhancing the sensitivity and resolution of the probe, and effectively broadening the bandwidth. Due to the thick vibration superposition of the piezoelectric wafers, the double-laminated ultrasonic transducer can generate higher energy.

The function of a plano-concave lens is to geometrically focus the emitted and received sound waves. Through the difference in sound velocity between the lens material and the medium, a refraction effect is produced, converging the sound wave energy into a specific area. This can significantly enhance the sound intensity and signal-to-noise ratio in this area, thereby effectively improving the resolution and penetration depth of ultrasonic signals.

### 2.2. The Material of the Transducer

In this experiment, piezoelectric materials are the core element determining the sensitivity of ultrasonic transducers. For this reason, PT piezoelectric ceramics are selected as the oscillator material for research. This material has a relatively high piezoelectric constant and electromechanical coupling coefficient, which is conducive to achieving high-sound electrical conversion efficiency. The matching layer and the backing layer are prepared by using tungsten powder–epoxy resin composite material with a tungsten powder mass fraction of 90%. This composite backing, with its high density and high sound attenuation characteristics, can effectively suppress ringing, shorten pulse width, thereby enhancing axial resolution and broadening bandwidth. Meanwhile, the high-matching layer helps to achieve impedance gradient matching between the piezoelectric wafer and the load, reducing the loss of sound wave reflection and improving the efficiency of sound energy transmission and system sensitivity. The two are optimized in synergy, aiming to enhance the comprehensive acoustic performance of the transducer. Table 1 shows the relevant parameters of the PT piezoelectric chip.

## 3. Simulation Analysis of Focused Laminated Transducers

**Figure 6:**
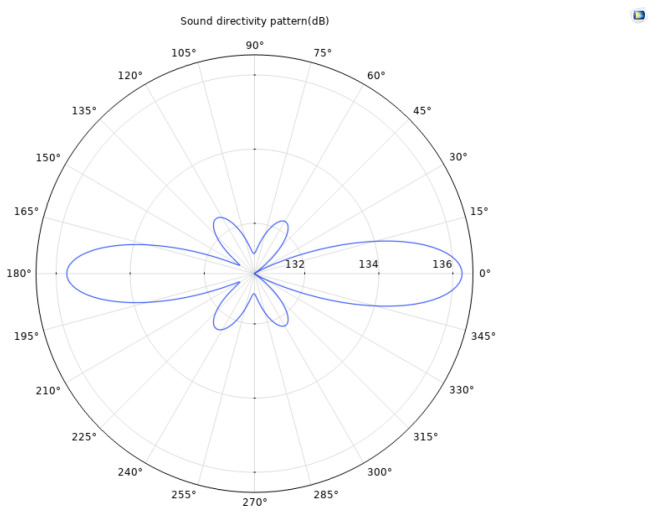
Directivity curve of focused single piezoelectric wafer ultrasonic transducer.

**Figure 7:**
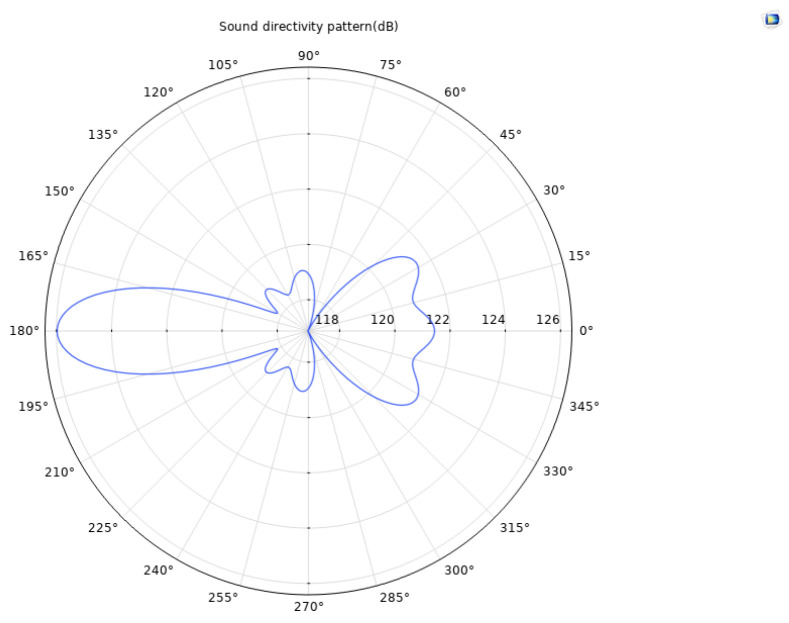
Directivity curve of the focused double-stack ultrasonic transducer.

Figure 6 and Figure 7 are, respectively, the schematic diagrams of the simulation models of single piezoelectric wafer and double laminated wafer transducers. The structure of this model mainly includes piezoelectric wafers, copper electrode layers, matching layers, plexiglass, and water. Among them, the piezoelectric wafer serves as the core driving unit; Copper sheets are distributed between the two wafers and are used to achieve electrical signal excitation and reception. The acrylic layer serves both acoustic-matching and protective purposes. Water medium is used to simulate the actual sound wave propagation environment. This model provides an effective simulation basis for analyzing the vibration characteristics and acoustic field performance of transducers.

**Figure 8:**
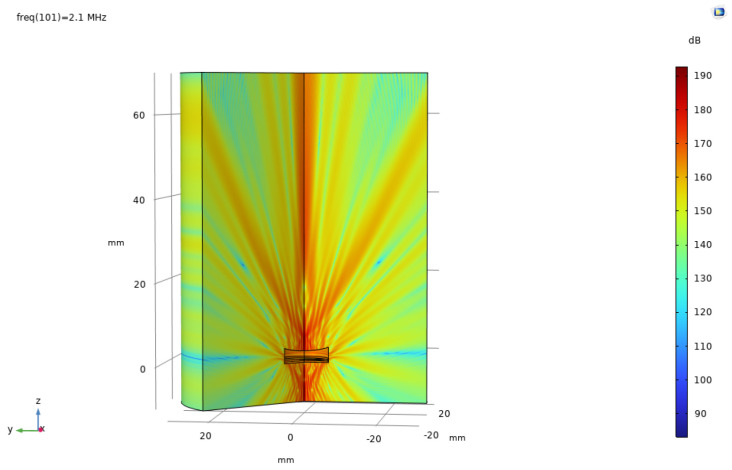
A simulation diagram of the sound pressure level of an ultrasonic transducer.

Based on the finite element method, a coupled numerical model including a solid mechanics module and an electrostatic module was established to simulate the acoustic field characteristics of the ultrasonic transducer. To accurately capture the propagation behavior of ultrasonic waves in the medium, the grid size is set to one-tenth of the ultrasonic wavelength, ensuring calculation accuracy while maintaining solution efficiency. After completing the transient or frequency domain solution, the sound field data was extracted and analyzed through the software post-processing function, and the far-field directivity distribution curve of the transducer was obtained. Meanwhile, a finite element model of the acoustic lens was established to analyze its focusing effect and its influence on the distribution of the sound field. Figure 6 and Figure 7, respectively, present the comparison results of the directivity curves of single piezoelectric chip and dual piezoelectric chip transducers under the same excitation conditions, which can visually reflect the differences in directivity characteristics such as beam width and side lobe level between the two, providing a basis for evaluating the performance of transducers and optimizing design. Figure 8 is a simulation diagram of the sound pressure when the ultrasonic transducer vibrates in water.

Based on the directivity curve data obtained from the simulation calculation, it can be analyzed that the far-field distance of the focused single piezoelectric wafer ultrasonic transducer is 17 mm. The directivity gain can be calculated according to the following formula:

(9) $$G_{d}=\frac{4\pi }{\int_{0}^{2\pi }\int_{0}^{\pi }{\left[D(\theta ,\phi )\right]}^{2}\sin\theta d\theta d\phi }$$

The directivity gain is 39.62 dB. Under the same structural conditions, the far-field distance of the focused dual piezoelectric wafer ultrasonic transducer is also 17 mm, with a directivity gain of 44.11 dB. The comparison shows that the directivity gain of the double-laminated transducer is 4.49 dB higher than that of the single-laminated piezoelectric transducer. This gain improvement reflects the significant advantages of the double-laminated structure in sound field control: its sound beam is narrower, energy is more concentrated, the main lobe width is effectively narrowed, and the side lobe level is better suppressed. This acoustic characteristic helps to enhance the echo signal strength and improve the signal-to-noise ratio, thereby demonstrating superior spatial resolution and defect recognition accuracy in actual detection, making it especially suitable for high-precision ultrasonic imaging and detection application scenarios.

## 4. Preparation and Performance Testing of Sensors

### 4.1. Electrode Wire Connection

A PT piezoelectric wafer with a frequency of 4 MHz was adopted. A tiny notch was fabricated on the wafer, and the wires were carefully welded onto the piezoelectric element at an appropriate welding temperature within the shortest possible welding time to ensure that the transducer had good performance. For this work, silver-plated aviation conductors with a diameter of 1 mm are selected for signal transmission. The size of the solder joints should be reduced as much as possible to minimize the impact on the transmitted and received sound fields. The length of the wire is usually 1 to 2 cm to facilitate subsequent performance tests.

### 4.2. Preparation of the Matching Layer

The matching layer uses epoxy resin and a curing agent as its base and is mixed with tungsten powder in a mass fraction of 90%. After thorough mixing to form a slurry, it is placed in a centrifuge for centrifugal treatment to remove air bubbles, ensuring the uniformity and density of the material. Subsequently, the mixed slurry and piezoelectric wafers are jointly loaded into a prefabricated mold and transferred to an infrared drying oven for curing and drying. The matching layer is processed to one-quarter of the ultrasonic wavelength to form a matching layer structure with stable acoustic performance.

### 4.3. Preparation of Backing Layer

The backing layer is prepared with epoxy resin–tungsten powder composite material of the same proportion (i.e., the mass fraction of tungsten powder is 90%). After removing the bubbles in the same way, the slurry is injected into the pre-prepared mold. The thickness of the prepared backing layer is approximately 20 mm. Subsequently, the backing layer is bonded to the opposite side of the piezoelectric wafer and the matching layer using the same type of slurry.

### 4.4. Acrylic Lens

In this experiment, a plano-concave lens made of organic glass was used for focusing. The diameter of the lens was 12 mm, which was equal to the diameter of the piezoelectric wafer, and the focal length of the lens was 30 mm.

### 4.5. Carbon Fiber Test Block

**Figure 9:**
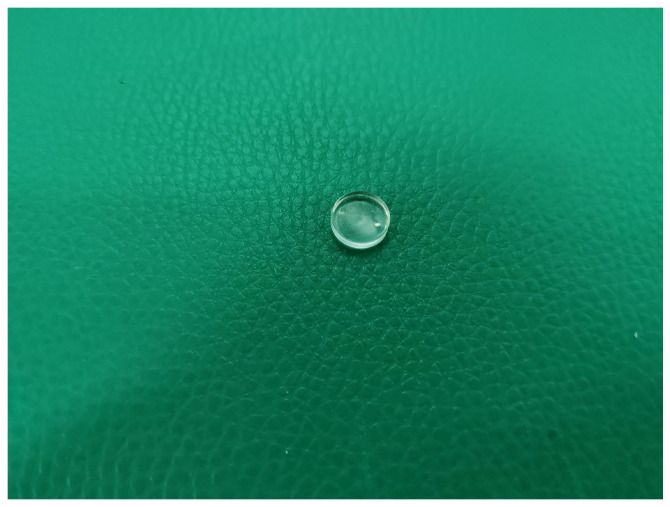
Real image of a plano-concave lens.

This test block uses 4mm thick T800 carbon fiber panels provided by Ximeng Hardware Tools Store in Zhejiang. According to the national standard GBT 27664.1-2011 [30] “Performance and Inspection of Non-destructive Testing Ultrasonic Testing Equipment”, a flat-bottom hole was prefabricated at a depth of 1 mm below the front of the plate to detect defects in carbon fiber composite materials. The fabricated carbon fiber plate is shown in Figure 9.

### 4.6. Testing Equipment

**Figure 10:**
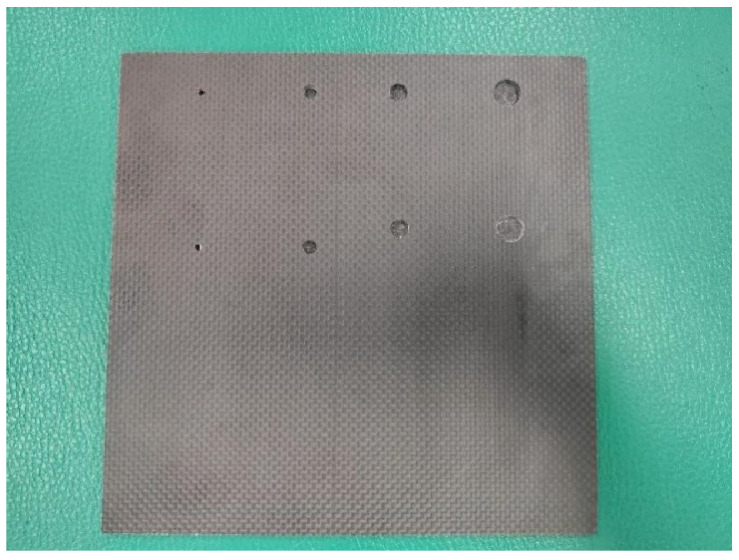
T800 carbon fiber test block drawing.

As shown in Figure 10, the test system is mainly composed of a water tank, a carbon fiber plate, a DPR300 pulse transceiver, and an oscilloscope. During the test, the prepared ultrasonic transducer is placed in the water tank for debugging. The DPR300 pulse transceiver emits pulses to excite the piezoelectric wafers in the transducer, causing them to vibrate and emit ultrasonic waves. The ultrasonic waves propagate to the carbon fiber plate and are reflected. The echo signal is received by the ultrasonic transducer, converted into an electrical signal by the piezoelectric wafer, and returned to the DPR300 pulse transceiver. Ultimately, the electrical signal is transmitted to the oscilloscope via the pulse transceiver for display and analysis.

### 4.7. Test Results

**Figure 11:**
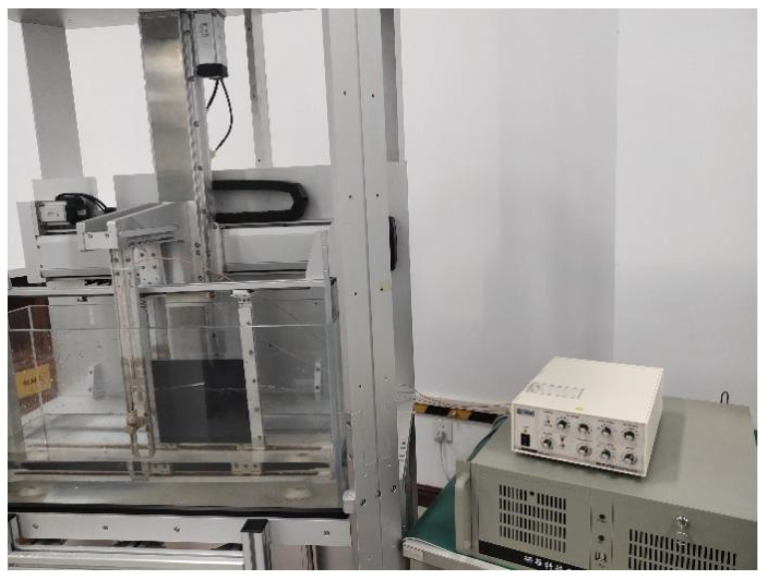
Test equipment diagram.

The fabricated ultrasonic transducer is shown in Figure 11. The fabricated ultrasonic transducer is placed perpendicularly to the carbon fiber and aimed at the defect area for inspection.

**Figure 12:**
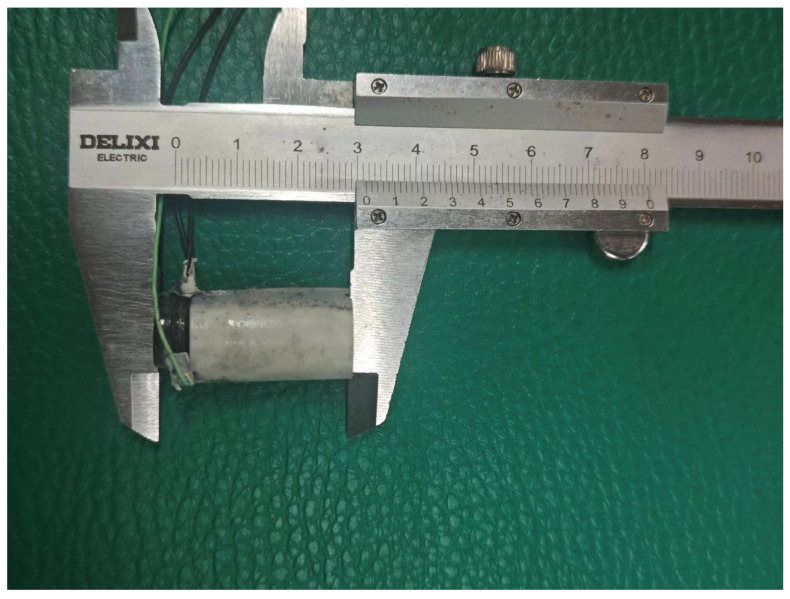
Transducer diagram.

**Figure 13:**
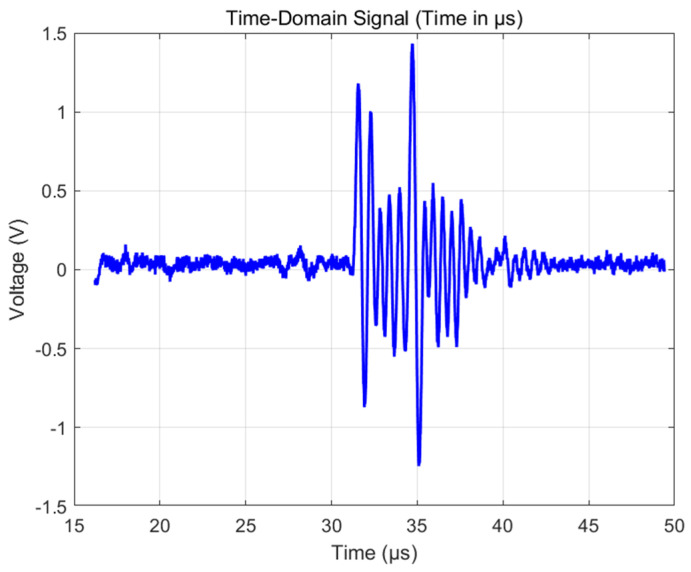
Echo signal of a double-laminated transducer.

**Figure 14:**
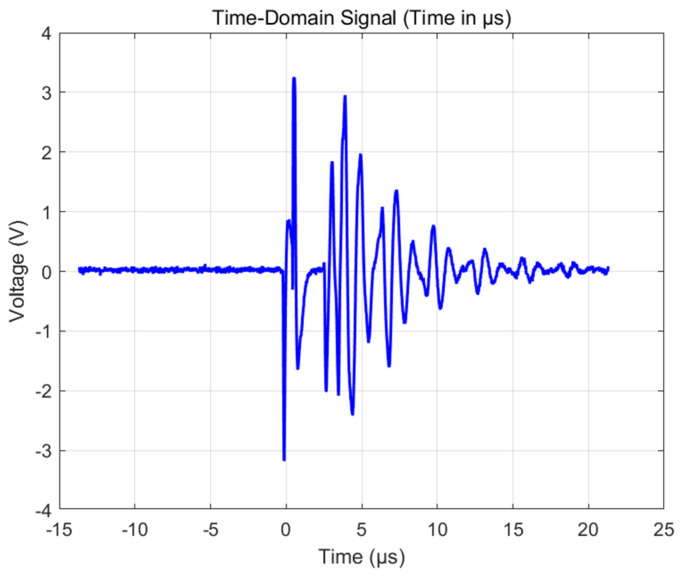
Standard contact transducer echo signal.

As shown in Figure 12, ultrasonic waves are reflected on the surface of the carbon fiber plate, generating the first echo signal with an amplitude range of −0.87 V to 1.17 V, and a sensor frequency of 1.88 MHz. After a delay of 3.2 µs, the sound wave was reflected back to the transducer through the defect, and the measured signal amplitude ranged from −1.24 V to 1.43 V. Based on the known sound velocity value of 3500 m/s in the carbon fiber composite material, the defect depth is calculated to be approximately 1 mm. Figure 13 and Figure 14 show the measurement results of the defective area by Olympus’ 1 MHz standard probe. The transducer detects that there is basically no difference between the defective area and the non-defective area, and, therefore, cannot accurately distinguish between the defects in carbon fibers. The result indicates that the developed ultrasonic transducer has successfully achieved the detection and depth positioning of internal buried defects in carbon fiber plates.

## 5. Conclusions

Based on the working principle of ultrasonic transducers, the following tasks have mainly been accomplished in this paper.

By setting the frequency range of the independent variable beyond the equation from 1999,950 Hz to 200,0050 Hz and solving it using the numerical diagram method, the resonant frequency of the double-laminated transducer was accurately obtained, which is 2 MHz. This method visually determines the frequency solution that satisfies the equation by drawing the function curve in a narrow frequency domain, thereby precisely characterizing the core vibration characteristics of the transducer.

The acoustic field characteristics of the transducer were modeled and numerically analyzed. The simulation results show that under the same structure and excitation conditions, the directivity gain of the double-laminated focused transducer is significantly improved compared with that of the single-component focused transducer, with a specific increase of 4.49 dB. This result indicates that the double-laminated structure effectively optimizes the concentration of acoustic energy and radiation efficiency, which is conducive to enhancing the detection sensitivity and spatial resolution ability of the transducer.

A focused ultrasonic transducer based on a double-laminated structure was fabricated and applied to the non-destructive testing of T800 carbon fiber composite materials. The experimental results show that this transducer can effectively identify and detect defects within the material, exhibiting excellent detection capability and signal-to-noise ratio characteristics, thereby verifying its practicality and reliability in the quality assessment of composite materials.

The results show that the focused laminated ultrasonic transducer can better enhance the sound field intensity, has a higher directivity gain, and exhibits good detection capability.

## Figures and Tables

**Table 1:** Key parameters of PT.

Parameter	PT
***d*_33_** **(** **×** **10** **^−^** **^12^** **C/N)**	352
***g*_33_** **(** **×** **10** **^−^** **^3^** **m^2^/C)**	18.7
***K_t_*** **(** **×** **10** **^−^** **^2^** **)**	53
***Y*_33_*^E^* (×10^10^N/m^2^)**	5.9
***T_c_*** **(°** **C** **)**	260
***ρ*** **(g/cm^3^)**	7.45
***σ***	0.32
***Q_m_***	65

## Data Availability

The raw data supporting the conclusions of this article will be made available by the authors on request.
